# Association of Long-Term Exposure to Fine Particulate Matter and Cardio-Metabolic Diseases in Low- and Middle-Income Countries: A Systematic Review

**DOI:** 10.3390/ijerph16142541

**Published:** 2019-07-16

**Authors:** Suganthi Jaganathan, Lindsay M. Jaacks, Melina Magsumbol, Gagandeep K. Walia, Nancy L. Sieber, Roopa Shivasankar, Preet K. Dhillon, Safraj Shahul Hameed, Joel Schwartz, Dorairaj Prabhakaran

**Affiliations:** 1 Centre for Chronic Disease Control, New Delhi 110016, India; 2 Department of Global Health and Population, Harvard T.H. Chan School of Public Health, Boston, MA 02115, USA; 3 Public Health Foundation of India, Gurgaon 122002, India; 4 Department of Environmental Health, Harvard T.H. Chan School of Public Health, Boston, MA 02115, USA; 5 London School of Hygiene and Tropical Medicine, London WC1E 7HT, UK

**Keywords:** air pollution, cardio-metabolic diseases

## Abstract

Background: Numerous epidemiological studies indicated high levels of particulate matter less than2.5 μm diameter (PM_2.5_) as a major cardiovascular risk factor. Most of the studies have been conducted in high-income countries (HICs), where average levels of PM_2.5_ are far less compared to low- and middle- income countries (LMICs), and their socio-economic profile, disease burden, and PM speciation/composition are very different. We systematically reviewed the association of long-term exposure to PM_2.5_ and cardio-metabolic diseases (CMDs) in LMICs. Methods: Multiple databases were searched for English articles with date limits until March 2018. We included studies investigating the association of long-term exposure to PM_2.5_ (defined as an annual average/average measure for 3 more days of PM_2.5_ exposure) and CMDs, such as hospital admissions, prevalence, and deaths due to CMDs, conducted in LMICs as defined by World Bank. We excluded studies which employed exposure proxy measures, studies among specific occupational groups, and specific episodes of air pollution. Results: A total of 5567 unique articles were identified, of which only 17 articles were included for final review, and these studies were from Brazil, Bulgaria, China, India, and Mexico. Outcome assessed were hypertension, type 2 diabetes mellitus and insulin resistance, and cardiovascular disease (CVD)-related emergency room visits/admissions, death, and mortality. Largely a positive association between exposure to PM_2.5_ and CMDs was found, and CVD mortality with effect estimates ranging from 0.24% to 6.11% increased per 10 μg/m^3^ in PM_2.5_. CVD-related hospitalizations and emergency room visits increased by 0.3% to 19.6%. Risk factors like hypertension had an odds ratio of 1.14, and type 2 diabetes mellitus had an odds ratio ranging from 1.14–1.32. Diversity of exposure assessment and health outcomes limited the ability to perform a meta-analysis. Conclusion: Limited evidence on the association of long-term exposure to PM_2.5_ and CMDs in the LMICs context warrants cohort studies to establish the association.

## 1. Background

Cardiovascular disease (CVD) is the leading cause of death in nearly all countries around the globe [1], and chronic exposure to air pollution is an important risk factor for CVD. Based on numerous epidemiological studies, particulate matter with a diameter of less than 2.5 micrometers (PM_2.5_) is considered to be the main culprit for these adverse cardiovascular effects [2,3,4]. Evidence from systematic reviews and meta-analyses suggests a strong association between short-term exposure to elevated PM_2.5_ and hospital admissions, myocardial infarction, stroke, and heart failure [5,6,7,8]. Even in countries where current international air quality standards are met (i.e., the World Health Organization (WHO) standards of 10 µg/m^3^ annual mean concentration or 25 µg/m^3^ 24-hour mean concentration) [2,9,10,11,12], small increases in PM_2.5_ are associated with increased risk of stroke [13]. 

More recent evidence suggests that chronic, i.e., long-term exposure to elevated PM_2.5_ also contributes substantially to cardio-metabolic diseases (CMDs) [4,14]. A meta-analysis from European and North American studies reported that the association between PM_2.5_ and type 2 diabetes mellitus (T2DM) increased the risk by 10% for every 10 µg/m^3^ increase in PM_2.5_ [15], which was similar to a meta-analysis conducted in 2014 that found an 11% increase in T2DM risk per 10 µg/m^3^ increase in PM_2.5_ among five identified studies [16]. Similarly, a systematic review conducted in 2015 identified five studies on long-term PM_2.5_ exposure and hypertension risk, and meta-analysis revealed a small albeit statistically non-significant effect summary odds ratio (OR) = 1.06 (95% confidence interval (CI), 0.98, 1.15) [17]. All of these reviews included only developed countries (US, Canada, and Europe).

Thus, while an association between PM_2.5_ and CMD has been demonstrated by epidemiological studies conducted in developed countries, where the annual average PM_2.5_ concentrations are near the WHO standard, scientific evidence from low- and middle-income countries (LMICs) is extremely limited [18]. This is concerning given substantially higher exposure levels in LMICs due to rapid industrialization and inadequate enforcement of environmental regulations [19]. Moreover, differences in the sources and composition of ambient air pollution, diet, and chronic disease status limit the generalizability of findings from developed countries [20]. To further define the current state of the science in LMICs, we conducted a systematic literature review of the association between long-term exposure to PM_2.5_ and CMDs in LMICs.

### Objective

To systematically compile evidence for the association between long-term exposure to PM_2.5_ and CMDs in LMICs.

## 2. Materials and Methods

We performed this study in three stages: database search, title and abstract screening, and full-text review and extraction. First, we searched the following databases: Medline and Embase (from January 1, 1948 to March 6, 2018), Cumulative Index to Nursing and Allied Health Literature, SCOPUS, Cochrane database, and Web of Science. We restricted our search to articles published in English, and we only considered peer-reviewed original articles. Detailed search terms are provided in Appendix A.

## 3. Definitions

For the purpose of this review, we defined CMDs as a clustering of disorders (hypertension, lipid disorders, hyperinsulinemia, and glucose intolerance) that together lead to CVD and T2DM [21,22]. We focused specifically on long-term exposure to PM_2.5_, defined as an annual average or average measure of more than 3 days of PM_2.5_ exposure. We chose to focus on long-term exposure, given that the diseases of interest (CMDs) are chronic diseases that develop over long periods of time. 

### 3.1. Inclusion and Exclusion Criteria

We included studies that:

(1) Were original research published in peer-reviewed journals up to March 6, 2018.
(2) Were conducted in an LMIC according to current World Bank classification [23].
(3) Quantified the association between exposure to PM_2.5_ and at least one CMD, including CVD, T2DM, lipid disorders, hypertension, hyperinsulinemia, and glucose intolerance, and related hospitalizations.
(4) Involved long-term exposure to PM_2.5_ (annual average or more than 3 days).

We excluded studies that:

(1) Were conducted only among the population aged less than 18 years.
(2) Used proxy measures to assess the exposure to PM_2.5_ (e.g., proximity to major roads).
(3) Focused on a specific population (e.g., patients with previous CVD events or T2DM).

### 3.2. Selection of Studies

The search strategy followed the PICO-population, intervention/exposure, comparator (there was no comparator in our review) and outcomes process [24]: population-general population from LMICs, intervention/exposure- long-term exposure to PM_2.5_, and outcomes - CMDs. Titles and abstracts were screened by three researchers (S.J., G.K.W., and M.M.). A full-text review was then performed independently by three researchers (S.J., G.K.W., and M.M). Disagreements were resolved by discussion in a small working group (S.J., G.K.W., M.M., and L.M.J.). Studies were then appraised for quality using the Cochrane Risk of Bias tool [25]. Each study was assessed for the following: selection bias, assessment of exposure and outcome, and adjustment for confounders. The studies were classified into three groups: high-quality (low risk of bias), medium-quality (unclear), and low-quality (high risk of bias). Two reviewers (S.J. and G.K.W.) independently appraised the studies, and disagreements were resolved in a consensus meeting. Only high-quality studies were included in this review.

### 3.3. Data Extraction

Data were extracted using a standardized extraction table that was piloted for two articles. Data extraction was performed by one researcher (S.J.) and was checked for accuracy by a small working group (G.K.W., M.M., and L.M.J.). The data extraction included study characteristics (author, year of publication, year of data collection, region, country, sampling method, sample size, location, setting), demographics (sex and age distribution), air pollution exposure information (method of measurement, length of exposure, and average measure), CMDs (prevalence/admissions/mortality, source of information), and results.

## 4. Results

### 4.1. Study Selection Process

**Figure 1:**
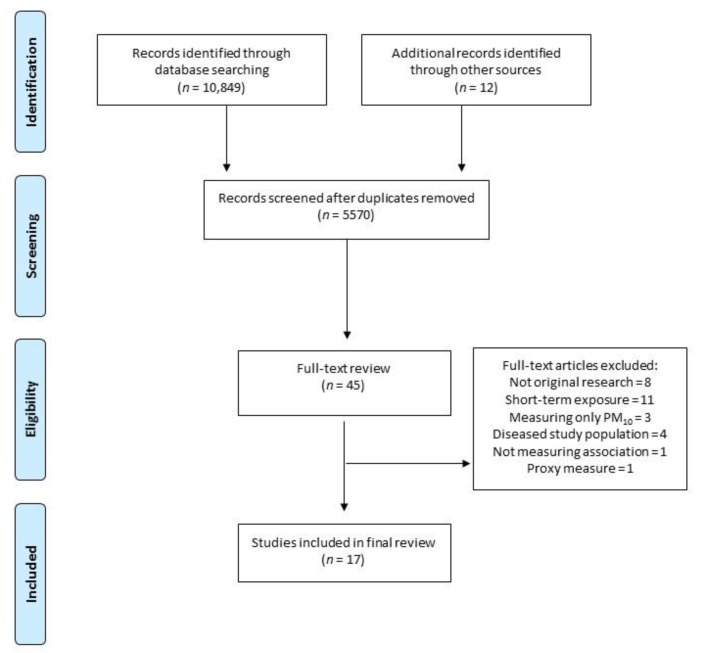
PRISMA flowchart [26].

We identified 10,861 articles in the database search, of which 5,291 were excluded as duplicates (Figure 1). Title and abstracts were screened, and 45 studies were identified for full-text review. Twenty-eight articles were excluded mainly because they were not original research (*n* = 8), did not assess PM_2.5_ (*n* = 3), assessed short-term PM_2.5_ exposure (*n* = 11), did not measure an association (*n* = 1), used proxy measures of PM_2.5_ exposure (*n* = 1), or were in patients with a pre-existing condition (*n* = 4). The final review included 17 studies (Table 1 and Table 2).

The following study designs were used in the included studies: seven were cross-sectional studies, eight were time-series studies, one was a time-stratified case-crossover study, and one was a cohort study. The included studies were conducted in the following countries: Brazil (*n* = 3), Bulgaria (*n* = 1), China (*n* = 11), India (*n* = 1), and Mexico (*n* = 1). Only two studies were conducted before 2008, and the rest were conducted after 2008 (past 10 years) on the studied outcomes.

### 4.2. Study Characteristics: Exposure and Outcomes

The most common methods of exposure assessment used in these studies were modeled estimates from hybrid space-time models, which use a combination of satellite remote sensing, meteorology, and land use as predictors (*n* = 8); data from nearby monitoring stations (*n* = 4); both direct measurements of PM_2.5_ and data from monitoring stations (*n* = 3) and direct measurement using personal monitors (*n* = 2). We could not perform meta-analyses due to the heterogeneity of PM_2.5_ measurement methods across studies. The average concentration of PM_2.5_ reported in all of the Chinese studies was 73.85 µg/m^3^ ranging from 33.7 µg/m^3^ to 147 µg/m^3^. PM_2.5_ concentrations reported by studies conducted in India (2015), Brazil (2010–2012), Bulgaria (2014), and Mexico (1995) were 136.9 µg/m^3^, 4.4 to 23.8 µg/m^3^, 66.8 µg/m^3^, and 27 µg/m^3^, respectively. With respect to CMD outcomes reported in the included studies, three studies evaluated hospital admissions and emergency room visits (ERV); nine studies evaluated CVD mortality; five studies evaluated CVD risk factors, including T2DM. Table 1 summarizes 11 studies which evaluated shorter PM_2.5_ exposure windows (3 days up to 40 days), and Table 2 summarizes six studies which evaluated longer PM_2.5_ exposure windows (annual average up to 13-year average). The exposure association to outcome grouped the included studies into two categories, namely studies measuring less than annual averages of exposure and studies measuring an annual average of exposure with an exact number of days of exposure assessment mentioned in the Table 1 and Table 2.

### 4.3. Mortality Outcomes

Of the nine studies that evaluated CVD mortality [27,31,33,35,36,37,38,43,44], eight (91%) reported significant effects of long-term PM_2.5_ exposure. Of these studies, six were conducted in China, and one study each was carried out in Mexico, India, and Brazil. For every 10 μg/m^3^ increase in PM_2.5_, CVD mortality increased, ranging from 0.24% (95% CI 0.05, 0.43) on 40th day after exposure to 6.11% (95% CI 1.76, 10.64) per interquartile (IQR) increase (31.5 μg/m^3^) in PM_2.5_ at moving averages for the previous 3 days [31,33].

The national prospective cohort study conducted in China, which included 189,793 men aged 40 years and above, reported a hazard ratio (HR) for CVD mortality for every 10 μg/m^3^ increase in PM_2.5_ of 1.09 (95% CI 1.08, 1.10) for a 6-year time period [43]. A time-series study from Guangzhou with a sample size of 33,721 adults reported that the excess risk (ER) of CVD mortality was 6.11% (95% CI 1.76, 10.64) per IQR increase in PM_2.5_ at moving averages for the previous 3 days [31]. A study of 145,477 adults aged 45 years and above conducted in Beijing reported the estimated percentage increase in the risk of death for every 10 µg/m^3^ increase in PM_2.5_. They found that the risk of CVD mortality rose by 0.24% (95% CI 0.05, 0.43), cerebrovascular disease (CBD) mortality by 0.23% (95% CI (-)0.03, 0.50), and ischemic heart disease (IHD) mortality by 0.22% (95% CI 0.06, 0.50) over 0–5 days [33]. For every 10 µg/m^3^ increase in PM_2.5_, a study in Shenyang, China found an increase in CVD mortality of 0.42% (95% CI 0.10, 0.73) [35]. In contrast, a study conducted in Chongqing, China found no association between CVD mortality and PM_2.5_ [37]. 

The only Mexican study reported that for every 10 μg/m^3^ increase in PM_2.5_, there was a 3.4% (95% CI 0.67, 6.18) increase in CVD mortality [27]. The only study from India reported a burden of 5700 (95% CI 2800, 7500) annual premature deaths (0.16% of the population) attributable to PM_2.5_ exposure, of which 29% and 18% were IHD and stroke, respectively. The estimated premature deaths per year from ambient PM_2.5_ exposure in Varanasi, India by IHD was 1600 (95% CI 600, 2200) and stroke was 1000 (95% CI 500, 1400) [38]. A Brazilian study reported that every 10 μg/m^3^ increase in PM_2.5_ resulted in a relative risk (RR) of 1.81% (95% CI 0.03, 3.61) for CVD mortality [36].

### 4.4. Hospital Admissions and Emergency Room Visits

There were four studies [29,32,34,36], one from China and three from Brazil that evaluated hospital admissions and ERV. All reported significant effects of long-term PM_2.5_ exposure. 

In Brazil, the RR was 2.64 (95% CI 1.60, 3.69) for hospitalizations related to PM_2.5_ over a period of 10 days [36]. Another study in Brazil reported a significant increase in the risk of hospitalization for circulatory system diseases of 19.6% (95% CI 6.4, 34.6) per 10 μg/m³ increase in PM_2.5_ 5 days after exposure [29]. The third study in Brazil similarly reported a 15% (SE 0.07%) increase in the risk of hospitalization for CVD per 10 µg/m^3^ increase in PM_2.5_ concentration 5 days after exposure [34].

The only Chinese study found that an IQR increase in PM_2.5_ (43.0 μg/m^3^) was associated with 0.3% (95% CI (-)2.4, 3.0) total CVD ERV for 11-day moving average [32].

### 4.5. CVD Risk Factors

Of the five studies that evaluated CVD risk factors [28,39,40,41,42], including T2DM, all reported significant effects of long-term PM_2.5_ exposure. Four of these studies were conducted in China, and one study was conducted in Bulgaria. The following CVD risk factors were assessed by studies included in this review: hypertension (*n* = 1), tachycardia and resting heart rate (*n* = 1), insulin resistance and T2DM (*n* = 3).

A study conducted in China among adults aged 50 years and above found that for every 10 μg/m^3^ increase in PM_2.5_ over a 3-year period, the adjusted OR for hypertension was 1.14 (95% CI 1.07, 1.22). Each 10 μg/m^3^ increase in ambient PM_2.5_ was associated with a 1.04 mmHg (95% CI 0.31, 1.78) increase in diastolic blood pressure and a 1.30 mmHg (95% CI 0.04, 3.56) increase in systolic blood pressure [39]. A cross-sectional survey among adults between 20 and 49 years, also from China over a 3-year period, reported, for every 10 μg/m^3^ increase in PM_2.5_, that the OR for tachycardia was 1.018 (95% CI 1.017, 1.020) and resting heart rate increased by 0.076 bpm (95% CI 0.073, 0.079) [41]. Another Chinese study found that PM_2.5_ was significantly associated with increased diabetes by OR: 1.14 (95% CI 1.03, 1.25) per IQR increase (26 μg/m^3^) in PM_2.5_ over a 3-year period [42]. 

A study in China among adults aged 45 years and above reported that an annual average IQR increase (41.1 μg/m^3^) in PM_2.5_ was significantly associated with increased T2DM prevalence, PR= 1.14 (95% CI 1.08, 1.20), elevated levels of fasting glucose by 0.26 mmol/L (95% CI 0.19, 0.32), and elevated HbA1c by 0.08% (95% CI 0.06, 0.10) [40]. A study from Bulgaria reported that PM_2.5_ was positively associated with T2DM, OR 1.32 (95% CI 0.28, 6.24) [28]. 

## 5. Discussion

We identified just 17 studies conducted in LMICs with the link between long-term PM_2.5_ exposure and CMD, and most of the studies (65% of studies) were in China. This is concerning because LMICs carry the greatest burden of both CMD and air pollution, and most of the studies we reviewed reported significant increases in CVD mortality with increasing PM_2.5_ levels. The Global Burden of Disease (GBD) in 2015 and Global Health observatory data repository reported that the population-weighted mean exposure to PM_2.5_ in China, India, Brazil, Mexico, and Bulgaria were 58.4 μg/m³ (95% CI 58.1, 58.7), 74.3 μg/m³ (95% CI 73.9, 74.8), 11.4 μg/m³ (95% CI 11.2, 11.5), 20.1 μg/m³ (95% CI 16.7, 27.2), and 18.8 μg/m³ (95% CI 18.3, 20.6), respectively. In our review, all the included studies indicated higher levels of PM_2.5_ than reported by the GBD except for one study from Brazil [29], which reported slightly lower estimates. In this review, PM_2.5_ estimates reported by Chinese studies ranged from 33.7 to 147 μg/m³; Indian PM_2.5_ estimates ranged from 94.4 to 136.9 μg/m³; Brazilian PM_2.5_ estimates ranged from 4.4 to 23.8 μg/m³; Mexican PM_2.5_ estimates was 27.0 μg/m³, and Bulgarian study reported 66.8 μg/m³, all of which were much higher than the values reported by the GBD study [4]. Therefore, the GBD estimates may be underestimating CVD deaths attributable to PM_2.5_ in the countries of focus. Similarly, a global review and meta-regression analysis also observed much lower levels of PM_2.5_ in China compared to our estimates [45]. Most (70%) of the research studies included in this review were conducted in major cities and industrial centers in China, including Beijing, Chongqing, Guangzhou, Liaoning, Pearl River Delta region, Shanghai, and Shenyang, which may at least partially explain this deviance from the GBD estimates. Satellite-based estimates must be used cautiously and following shortcomings must be kept in mind: underestimation of ground measurements in locations with higher concentrations like East Asia, South Asia, North Africa, and Sub-Saharan Africa [46].

The duration of exposure ascertainment to outcome varied from a 3-day average to a 13-year average. The effect estimates varied with duration of exposure- longer exposure periods associated with smaller effect estimates. We observed differences among the effect estimates for cardiovascular mortality for annual averages or longer duration and less than annual averages. In our review, we observed that the effect estimates were smaller when the exposure window was longer, which needs further investigation. This was similar to a global meta-analysis of cohort studies, which also found that the effect estimates decreased with increasing concentrations [45]. 

This review was focused on CMDs as we currently lack literature on the effects of long-term exposure to PM_2.5_ on CMDs in LMIC settings. Globally, 17.1% of IHD mortality and 14.2% of CBD mortality in 2015 was attributable to PM_2.5_ as reported by GBD [7]. A prospective European cohort study found an increased risk of stroke associated with a 5 μg/m^3^ increase in PM_2.5_ with a hazard ratio of 1.19 (95% CI 0.88, 1.62) [13]. In our review, Luo et al. from their Beijing study reported that every 10 µg/m^3^ increase in PM_2.5_ was associated with an increased risk of CBD mortality of 0.23% (95% CI (-)0.03, 0.50) [33].

Across all of the included studies, the results ranged from 0.24% [33] to 6.11% [31] increase in CVD mortality for every 10 μg/m^3^ increase in PM_2.5_. While this effect estimate is relatively small, the ubiquitous nature of the exposure is likely to result in a large population-attributable disease burden. This range of values was higher than a recent meta-regression analysis of 53 studies, that found the percent increase at mean exposure of 10 μg/m^3^ PM_2.5_ associated with a 1.46% (95% CI 1.25, 1.67) increase in CVD mortality [45]. Similarly, a review of European epidemiological studies reported that the relative risk of CVD mortality was 1.11 (95% CI 1.07, 1.15) for a quartile increase in PM_2.5_ [47]. Another global review also noted that cardiorespiratory mortality was increased by 2.29% (95% CI 1.36, 3.85) per 10 µg/m^3^ increase in PM_2.5_ [48]. The effect estimates depended on the type of exposure assessment method as using ground-level monitors produced estimates 44–46% lower than that of hybrid space-time model [45]. Most of the studies reviewed here utilized secondary data from stationary monitoring stations (seven studies) or were modeled estimates (eight studies), which might not accurately reflect personal exposures. The only Indian study reported that premature mortality per year in the city of Varanasi from IHD was 1600 (95% CI 600, 2200) and stroke was 1000 (95% CI 500, 1400) after exposure to PM_2.5_ that could be saved [38]. There were no Indian studies measuring the impact of ambient air pollution on cardio-metabolic health outcomes, which needs focus in the future studies, given the fact that air pollution levels are alarmingly high, especially in the Indo-Gangetic plain [49].

A strong association between PM_2.5_ and CVD hospital admissions was observed. A pooled estimate from European countries reported that relative risk of cardiovascular hospitalizations increased by 1.8% (95% CI 0.1, 3.4) per IQR increase in PM_2.5_ (12.4 µg/m^3^) [50]. Results from a global review and meta-analysis showed that hospital admissions due to cardiorespiratory diseases were increased by 1.64% (95% CI 1.06, 2.53) per 10 µg/m^3^ increase in PM_2.5_ [48], which was much lower than the studies from Brazil identified in this review that reported a 15–19.6% increase in CVD hospitalizations [29,34]. On the contrary, a Chinese study observed that total and severe CVD ERVs increased by 0.3% (95% CI (-)2.4, 3.0) and decreased by 0.1% (95% CI 3.4, 3.3) for an IQR increase in PM_2.5_ by 43.0 μg/m^3^ in 11-day moving average, respectively, which indicated delayed association [32]. The varying effect estimates could be attributed to the population included and the exposure assessment method used in these studies.

Long-term exposure showed the strongest associations with hypertension. In a meta-analysis, there was a statistically insignificant increase in hypertension risk (OR = 1.065, 95% CI 0.985–1.152) with each 10 µg/m^3^ increment in PM_2.5_ [17]. We found very similar results from the Chinese study by Lin et al. who reported an increased risk of hypertension by an OR = 1.14 (95% CI 1.07, 1.22) with each 10 µg/m^3^ increment in PM_2.5_ [39]. A meta-analysis among cohort studies reported a positive association for T2DM and relative risk of 1.25 (95% CI 1.10, 1.43) for every 10 μg/m^3^ increase in PM_2.5_. Another review of European and North American studies reported that the pooled relative risk estimate for T2DM per 10 μg/m^3^ increase in PM_2.5_ was 1.10 (95% CI 1.02, 1.18) [15]. The Chinese study from our review reported that for an IQR increase in PM_2.5_, T2DM prevalence increased by PR = 1.14 (95% CI 1.08, 1.20) [40]. There were no studies conducted on lipid disorders with respect to air pollution exposure.

In this review, about two-thirds of the studies included were conducted in the last three years (2016–2018) indicating that this area is gaining more focus recently. A prospective cohort study design is considered the gold standard, but only one study, which was conducted in China, used a cohort design [43]. Further studies examining personal exposure to PM_2.5_ or statistically modeled estimates for PM_2.5_ and CMDs in LMICs are in urgent need to help develop scientific literature. 

This study is not without its limitations, firstly the heterogeneity of identified studies prohibited the use of meta-analytical methods to produce summary estimates of effect. We focused on long-term exposures considering that in LMICs, ambient air pollution is chronic exposure and effects may be larger for long-term exposures versus short-term exposures. From our review results, we cannot conclude this assumption, as we found that for a longer duration of exposure, the effect estimates were smaller, and further investigation is needed to confirm this. The second limitation is that we focused solely on one component of ambient air pollution, PM_2.5_, yet individuals are exposed to complex mixtures. We chose to focus on PM_2.5_ versus PM_10_ because previous research has demonstrated a stronger effect on CVD due to the ability of PM_2.5_ particles to travel to distant organs [14]. Moreover, most included studies used a single-pollutant model, but a few also measured other pollutants, including PM_10_, PM_1_, sulfur dioxide (SO_2_), nitrogen dioxide (NO_2_), nitrous oxide (NO), carbon monoxide (CO), ozone (O_3_), black carbon, organic and elemental carbon, soluble ions, and noise and traffic pollution. We evaluated only the effect estimates of PM_2.5_ here. The third limitation is the type of studies included in this review is very different and, hence, the effect sizes should be interpreted with caution. Future research should expand to include multipollutant models that take into account potential interactions between pollutants, as well as source-specific effects.

In conclusion, few studies have evaluated the association between long-term exposure to PM_2.5_ and CMD outcomes in LMICs, and the majority of this literature has come from China. We did not identify a single study conducted in North and Sub-Saharan Africa, which is home to 17% of the world’s population. Considering that the vast majority of morbidity and mortality attributable to PM_2.5_ is in LMICs, this represents a major environmental injustice. The global environmental health community must drive a strong research agenda relating to the CMD effects of PM_2.5_ in these settings in order to provide context-specific evidence to policymakers in these countries. 

## Figures and Tables

**Table 1:** Study characteristics, exposure, outcome, and primary results of included studies (*n* = 11, listed alphabetically) measuring PM_2.5_ exposure less than the annual average.

Citation	Place of Study	Study Period	Study Design	Study Population (Age, Gender)	Sample Size	Particulate Matter Measuring Less Than 2.5 µm (PM_2.5_) Measurement Method	Mean PM_2.5_ μg/m^3^	Exposure Association to Outcome (No. of Days Exposure Data- Available)	Outcomes (Source)	Results
[27]	Mexico City	1993–1995	Time-series study	Residents aged 65 years and above	4129	Monitoring station with 24-hour integrated particle mass	27	4 days (941 days)	Daily mortality (Electronic records)	Every 10 μg/m^3^ increase in PM_2.5_ was associated with a 3.4% (95% confidence interval (CI) 0.67, 6.18) increase in cardiovascular disease (CVD) mortality
[28]	Plovdiv, Bulgaria	2014	Cross-sectional survey	General population aged 18 years and above	513	Official municipality source	66.8	Not Available (NA) (150 days)	Type 2 diabetes mellitus (T2DM) prevalence (survey)	T2DM was positively associated with PM_2.5_ : Odds Ratio(OR) 1.32 (95% CI 0.28, 6.24) comparing top quartile (25.0–66.8 μg/m^3^) to bottom quartile (0.0–25.0 μg/m^3^)
[29]	São José dos Campos, Brazil	2010–2011	Time-series study	Daily hospital admissions in elderly people (60 years and above)	1765	Gent Stacked Filter-like sampler; Companhia Ambiental do Estado de São Paulo—CETESB monitoring station	4.4	5 days (350 days)	CVD hospital admissions (Health Services Information Database)	Every 10 μg/m³ increase in PM_2.5_ increased the risk of CVD hospitalization by 19.6% (95% CI 6.4, 34.6)
[30]	Six cities of the Pearl River Delta region, China	2013–2015	Cross-sectional survey	Deaths registered	316,305	Monitoring stations in each of the six cities: Guangzhou, Shenzhen, Zhuhai, Dongguan, Foshan, and Jiangmen	35.1 to 47.9	4 days (886 days)	CVD Mortality (Guangdong Provincial Center for Disease Control and Prevention)	Every 10 μg/m^3^ increase in PM_2.5_ concentration was associated with 2.19% (95% CI 1.80, 2.59) increase in CVD mortality
[31]	Guangzhou, China	2007–2011	Time-series study	General population	33,721	Panyu Meteorological Center, South China Institute of Environmental Sciences & GRIMM Aerosol Spectrometer	41.4	4 days (1079 days)	CVD mortality (Guangdong Provincial Center for Disease Control and Prevention)	An inter-quartile range (IQR) increase in PM_2.5_ (31.5 μg/m^3^) was associated with excess risk of CVD mortality by 6.11% (95% CI 1.76, 10.64)
[32]	Beijing, China	2004–2006	Time-series study	Emergency room visits (ERV)	13,026	Twin Differential Mobility Particle Sizer and Aerodynamic Particle Sizer	109.8	11 days (1035 days)	CVD ERV (Emergency Department of Hospital)	An IQR increase in PM_2.5_ (43.0 μg/m^3^) was associated with 0.3% (95% CI (-)2.4, 3.0) and (-)0.1 (95% CI 3.4, 3.3) total and severe CVD ERV, respectively
[33]	Beijing, China	2008–2011	Time-stratified case-crossover study	Death registered	145,477	U.S. embassy	95.7	40 days (1046 days)	CVD/ Cerebrovascular disease (CBD)/Ischemic Heart Disease (IHD) (Death Registry of Chinese Center for Disease Control and Prevention)	Every 10 µg/m^3^ increase in PM_2.5_ was associated with an increased risk of CVD mortality of 0.24% (95% CI 0.11, 0.39), CBD mortality of 0.23% (95% CI 0.03, 0.42), and IHD mortality of 0.22% (95% CI 0.12, 0.54)
[34]	São José do Rio Preto, Brazil	2011–2012	Time-series study	Hospitalizations registered	4505	Coupled Chemistry Aerosol-Tracer Transport model to the Brazilian developments on the Regional Atmospheric Modeling System (CCATT BRAMS model)	23.8	5 days (365 days)	CVD hospitalization (Unified Health System)	Every 10 µg/m^3^ increase in PM_2.5_ was associated with a 15% increased relative risk (RR) for CVD hospitalization with SE of 0.007%
[35]	Shenyang, China	2006–2008	Time-series study	General population	32	Continuous monitoring system at Shenyang Regional Meteorological Center; Ambient Dust Monitor 365	95.9	3 days (730 days)	Cause specific mortality (Liaoning Provincial Center for Disease Control and Prevention)	Every 10 µg/m^3^ increase in PM_2.5_ was associated with a 0.42% (95% CI 0.10, 0.73) increase in CVD mortality
[36]	Cuiabá and Várzea Grande, State of Mato Grosso, Brazil	2009–2011	Time-series study	General population aged 45 years and above	8610 hospitalizations and 3024 deaths	Method developed and validated for the Brazilian Amazon and Cerrado	17.7	10 days (983 days)	Daily mortality and hospitalization (Mortality Information System and Hospital Information System)	Every 10 μg/m^3^ increase in PM_2.5_ was associated with a 2.64% (95% CI 0.53, 4.06) increase in risk of CVD hospitalizations and 3.57% (95% CI 0.82, 6.38) increase in CVD mortality
[37]	Chongqing, China	1995	Cross-sectional survey	Deaths registered	47	24-hour samples collected from two roadside sites from representative areas of differing principal social activities	147	5 days (213 days)	Daily mortality (Chongqing Anti-Epidemic Station)	Every 100 μg/m^3^ increase in PM_2.5_ was associated with 1.09% (95% CI 0.95,1.20) increase in CVD mortality

CI-confidence interval; CVD-cardiovascular disease; PM-Particulate matter; NA-not available; T2DM-type 2 diabetes mellitus; OR-odds ratio; IQR-inter-quartile range; IHD-ishcemic heart disease; CBD-cerebrovascular disease; RR-relative risk.

**Table 2:** Study characteristics, exposure, outcome, and primary results of included studies (*n* = 6, listed alphabetically) measuring PM_2.5_ exposure as annual average or more.

Citation	Place of Study	Study Period	Study Design	Study Population (Age, Gender)	Sample Size	PM_2.5_ Measurement Method	Mean PM_2.5_ μg/m^3^	Exposure Association to Outcome (No. of Days Exposure Data-Available)	Outcomes (Source)	Results
[38]	Varanasi, India	2001–2015	Time-series study	General population	5700	MODIS onboard NASA-EOS AQUA and TERRA satellites	136.9	13 years (13 years)	Premature mortality from ischemic heart disease (IHD), Stroke (Premature deaths)	The estimated premature deaths per year from ambient PM_2.5_ exposure in Varanasi: IHD 1600 (95% CI 600, 2200), Stroke 1000 (95% CI 500, 1400)
[39]	Shanghai and seven provinces of Guangdong, Hubei, Jilin, Shaanxi, Shandong, Yunnan, and Zhejiang, China	2007–2010	Cross-sectional survey	General population aged 50 years and above	12,665	van Donkelaar and co-workers to estimate the outdoor PM_2.5_ concentrations	33.7	3 years (3 years)	Hypertension (World Health Organistion (WHO) Study on global aging and adult health)	Odds ratio (OR) for hypertension: 1.14 (95% CI 1.07, 1.22) for every 10 μg/m^3^ increase in PM_2.5_
[40]	National, China	2011–2012	Cross-sectional survey	General population aged ≥45 years	11,847	Satellite-based spatial statistical model	72.6	1 year (303 days)	Type 2 diabetes mellitus (T2DM) prevalence, blood glucose, and HemoglobinA1c (HbA1c) (survey)	An inter-quartile range (IQR) increase in PM_2.5_ (41.1 μg/m^3^) was associated with increased T2DM prevalence ratio( PR)1.14 (95% CI 1.08, 1.20), elevated levels of fasting glucose by 0.26 mmol/L (95% CI 0.19, 0.32), and HbA1c by 0.08% (95% CI 0.06, 0.10)
[41]	National, China	2015	Cross-sectional survey	General population aged 20–49 years	10,843,140	Hybrid geophysical statistical approach	47.1	3 years (3 years)	Tachycardia and Resting heart rate (Survey)	OR for tachycardia: 1.018 (95% CI 1.017, 1.020) and a 0.076 (95% CI 0.073, 0.079) bpm elevation in the resting heart rate for every 10 μg/m^3^ increase in PM_2.5_
[42]	Liaoning, China	2009	Cross-sectional survey	General population aged 18–74 years	15,477	Satellite-based spatial statistical model	82.0	3 years (3 years)	Prevalence of diabetes, fasting glucose, 2-hour glucose, and 2-hour insulin (Survey)	An IQR increase in PM_2.5_ (26 μg/m^3^) was significantly associated with increased diabetes: OR 1.14 (95% CI 1.03, 1.25)
[43]	National, China	2000–2005	Cohort study	Males 40 years and above	189,793	Combination of satellite-derived and chemical transport model estimates calibrated to surface measurements	43.7	6 years (6 years)	CVD Mortality (Survey)	Hazard ratio (HR) for CVD mortality was 1.09 (95% CI 1.08, 1.10) for every 10 μg/m^3^ increase in PM_2.5_

CBD-Cerebrovascular Disease; CI-Confidence Interval; CVD-Cardiovascular Diseases; DBP-Diastolic Blood Pressure; T2DM-Type 2 Diabetes Mellitus; ER-Excess Risk; ERV-Emergency Room Visits; HbA1c-HemoglobinA1c; HR-Hazard Ratio; IHD-Ischemic Heart Disease; IQR-Interquartile Range; OR-Odds Ratio; PM-Particulate Matter; PR-Prevalence ratio; RR-Relative Risk; SBP-Systolic Blood Pressure.

## Abbreviations

CBD-Cerebrovascular Disease;
CI-Confidence Interval;
CMD-Cardio-Metabolic Diseases;
CO-Carbon Monoxide;
CVD-Cardiovascular Diseases;
DBP-Diastolic Blood Pressure;
DM/T2DM-Diabetes Mellitus/Type 2 Diabetes Mellitus;
ER-Excess Risk; ERV-Emergency Room Visits;
HbA1c-HemoglobinA1c;
HIC-High Income Countries;
HR-Hazard Ratio;
IHD-Ischemic Heart Disease;
IQR-Interquartile Range;
LMIC-Low-and Middle-Income Countries;
NO_2_-Nitrogen Dioxide;
NO-Nitrous Oxide;
O_3_-Ozone; OR-Odds Ratio;
PM-Particulate Matter;
RR-Relative Risk;
SBP-Systolic Blood Pressure;
SD-Standard Deviation;
SO_2_-Sulfur Dioxide;
WHO-World Health Organization.

## Appendix A

### Appendix A.1. Search Terms

Air pollution: "air pollution" or "particulate matter" or "air pollutants" or "ambient air pollution" or "Total suspended particles" or "particulate matter" or "soot"

CMD: "diabetes" or "diabet*" or "IDDM" or "NIDDM" or "Cardiovascular*" or "Cardiovascular Diseases" or "Cardiovascular Abnormalities" or "Heart Arrest" or "Cardiac arrest*" or "Arterial Occlusive Diseases" or "Cerebrovascular Disorders" or "Hypertens*" or "Myocardial Ischemia" or "Prehypertens*" or "Angina*" or "Glucose Metabolism Disorders" or "Diabetes*" or "Prediabet*" or "Hyperglycem*" or "Glucose Intoleran*" or "Insulin Resistan*" or "Lipoprotein*" or "Apolipoprotein*" or "Chylomicrons" or "Chylomicron Remnants" or "Cholesterol*" or "High-Density Lipoproteins, Pre-beta" or "Cardiac Death" or "Asphyxia" or "Brain Death" or "Death, Sudden" or "Death, Sudden, Cardiac" or "Sudden Cardiac Death" or "Sudden Cardiac Arrest" or "Cardiac Arrest, Sudden"

Countries: Low- and Middle- Income Countries (Low income, lower middle income, upper middle-income countries) and “NOT” High Income Countries (see Table A1 for detailed search strategy).

### Appendix A.2. Cochrane Risk of Bias Tool

**Table A1:** Low risk of bias.

	Study Title	Selection	Exposure Assessment	Confounders	Outcome Assessment
1	Borja-Aburto 1998				
2	Dzhambov 2016				
3	Ferreira 2016				
4	Jain 2017				
5	Lin 2016				
6	Lin 2016				
7	Lin 2017				
8	Liu 2013				
9	Liu 2016				
10	Luo 2016				
11	Mantovani 2016				
12	Meng 2013				
13	Rodrigues 2017				
14	Venners 2003				
15	Xie 2018				
16	Yang 2018				
17	Yin 2017				
